# Human serum albumin: prediction model and reference values for preterm and term neonates

**DOI:** 10.1038/s41390-024-03634-1

**Published:** 2024-10-11

**Authors:** Zoë Vander Elst, Annouschka Laenen, Jana Deberdt, Lotte Delemarre, Pieter Vermeersch, Glynis Frans, Gunnar Naulaers, Matthias Gijsen, Erwin Dreesen, Isabel Spriet, Karel Allegaert, Anne Smits

**Affiliations:** 1 Department of Development and Regeneration, KU Leuven, Leuven, Belgium; 2 Neonatal Intensive Care Unit, University Hospitals Leuven, Leuven, Belgium; 3 Leuven Biostatistics and Statistical Bioinformatics Centre (L-Biostat), KU Leuven, Leuven, Belgium; 4 Faculty of Medicine, KU Leuven, Leuven, Belgium; 5 Department of Laboratory Medicine, University Hospitals Leuven, Leuven, Belgium; 6 Department of Pharmaceutical and Pharmacological Sciences, KU Leuven, Leuven, Belgium; 7 Pharmacy Department, UZ Leuven, Leuven, Belgium; 8 Department of Hospital Pharmacy, Erasmus MC, Rotterdam, The Netherlands

## Abstract

**Background:**

Human serum albumin (HSA) concentrations may alter HSA-bound drug distribution. This study aims to describe longitudinal real-world HSA trends, and to develop a prediction model for HSA concentrations using a large neonatal cohort.

**Methods:**

Patients admitted to the neonatal intensive care unit of the University Hospitals Leuven (postnatal age (PNA) ≤28days) were retrospectively included. Using linear mixed models, covariate effects on HSA were explored. A multivariable prediction model was developed (backward model selection procedure, 1% significance level).

**Results:**

In total, 848 neonates were included [median(interquartile range) gestational age (GA) 35(32–38)weeks, birth weight (BW) 2400(1640–3130)grams]. Median HSA concentration was 32.3(28.7–35.6)g/L. Longitudinal analyses demonstrated increasing HSA concentrations with PNA and GA for most GA groups. Univariable analyses revealed significant associations of HSA with PNA, GA, BW, current weight, total and direct bilirubin, total plasma proteins, respiratory support, mechanical ventilation, sepsis, ibuprofen use, and C-reactive protein (*p*-values < 0.05). A high-performance (R^2^ = 76.3%) multivariable HSA prediction model was developed, and PNA- and GA-dependent HSA centiles were provided.

**Conclusion:**

Population-specific HSA centiles and an accurate neonatal HSA prediction model were developed, incorporating both maturational and non-maturational covariates. These results can enhance future clinical care and pharmacokinetic analyses to improve pharmacotherapy of HSA-bound drugs in neonates, respectively.

**Impact:**

- To improve future pharmacokinetic modeling initiatives, a high-performance human serum albumin (HSA) prediction model was developed for (pre)term neonates, using a large, single-center cohort of real-world data.
- This prediction model integrates both maturational and non-maturational covariates, resulting in accurate HSA predictions in neonates.
- Additionally, HSA centiles based on postnatal and gestational age were developed, which can be easily applied in clinical practice when interpreting HSA concentrations of neonates.
- In general, unbound drug fractions are higher in neonates compared to older populations. To improve pharmacotherapy of HSA-bound drugs in neonates, the obtained results can be integrated in future pharmacokinetic-pharmacodynamic analyses.

## Introduction

Human serum albumin (HSA) is the most abundant plasma protein, comprising 50–60% of total plasma proteins and accounting for 57–80% of the plasma colloid osmotic pressure.^1–4^ HSA binds and transports endogenous and exogenous molecules in the bloodstream, such as free fatty acids (FFA), bilirubin and drugs.^1,4^ Moreover, HSA plays a role in the acid-base balance, has anticoagulant effects, and exhibits antioxidant activity.^4^

HSA concentrations are influenced by maturational (e.g. age) and non-maturational (e.g. critical illness) factors.^1,5^ Concentrations are lower in neonates compared to older populations, and lowest in preterm neonates due to liver immaturity resulting in lower HSA synthesis and absence of placental transfer.^3,6–8^ Concentrations of HSA increase with postnatal (PNA) and gestational age (GA).^5–7^ However, concentrations decrease during critical illness due to decreased gene transcription and capillary leakage.^1^ Lower HSA concentrations in preterm or critically ill neonates may have harmful effects, like potentiating bilirubin neurotoxicity risk due to fewer bilirubin binding places.^9^ Additionally, low HSA concentrations have been associated with worse outcomes in neonates undergoing cardiac surgery.^10,11^

Due to its drug-binding and transporting functions, HSA influences the distribution of HSA-bound drugs.^12^ Overall, neonates have higher unbound drug fractions compared to older populations as a result of lower HSA concentrations and a possibly altered HSA binding affinity.^12,13^ Moreover, competing endogenous substances for HSA binding sites, such as FFA and bilirubin, can cause drug displacement, increasing unbound drug fractions in neonates.^12,13^ The unbound drug is pharmacologically active and can result in drug (side-)effects.^14^

To understand and predict the impact of HSA concentrations on drug exposure (pharmacokinetics, PK) and drug (side-)effects (pharmacodynamics, PD), population-specific HSA reference ranges and their covariates are needed. Several studies have tried to establish neonatal HSA reference ranges, although most studies are based on limited data. We are aware of three other neonatal HSA prediction functions in literature.^15–17^ However, these are rather simple functions only based on the maturational covariates postmenstrual age (PMA) or PNA, and not validated. To fully capture neonatal physiology in PK modelling analyses, more accurate HSA trends and predictions are needed. Based on these knowledge gaps, the aims of this study are to describe longitudinal trends in real-world HSA concentrations of a large neonatal cohort, to develop population-specific HSA centiles for application in clinical care, and to develop a multivariable prediction model for HSA in preterm and term neonates. This prediction model can be integrated in future PK modeling initiatives to improve pharmacotherapy of HSA-bound drugs in neonates.

## Materials and methods

### Study design and patients

This study was performed at the neonatal intensive care unit (NICU) of the University Hospitals Leuven (UZL), Belgium, which is a level IV NICU taking care of (extremely) preterm and term neonates, including neonates with infections or perinatal asphyxia, and providing specialized surgical care including cardiac surgery. This NICU also acts as a referral center for (complex) congenital malformations, e.g. congenital diaphragmatic hernia and spina bifida. Neonates admitted to the UZL NICU between June 2015 and April 2017 were included in this retrospective study if they had a PNA of ≤28 days and at least one HSA determination available. Measurement of HSA is often performed in the standard of care blood sampling in our unit. We longitudinally extracted all HSA concentrations (g/L) from routine blood laboratory reports. In addition, following clinical characteristics were collected: PNA (days), GA (weeks), PMA (weeks), birth weight (BW, grams), current weight (CW, grams), sex (male/female), admission diagnosis as reported in the discharge report, sepsis (yes/no, both early- and late-onset), total and direct bilirubin (mg/dL), and total plasma proteins (g/L). Gestational age and PMA were reported in full weeks. Day of birth was defined as day 1. Sepsis was divided into suspected and confirmed sepsis. Suspected sepsis was defined by the treating clinician, as part of routine clinical care. Confirmed sepsis was defined by the isolation of a pathogen from a blood culture. To assess disease severity, information on respiratory support (yes/no), mechanical ventilation (yes/no), ibuprofen use (yes/no), serum creatinine (mg/dL) and C-reactive protein (CRP, mg/L) at the time of HSA measurement were collected.

### Measurements of laboratory values

Arterial or venous blood samples for HSA determination were collected in Minicollect Lithium heparin tubes (Greiner Bio-One, Kremsmünster, Austria) and analyzed at the UZL Department of Laboratory Medicine (Herestraat 49, 3000 Leuven, Belgium). Samples were stored at room temperature until arrival at the laboratory. Subsequently, samples were centrifuged for 10 min at 2000 x *g*, and analyzed by a colorimetric assay with bromocresol green (ALB2 reagent, reference number: 05166861) on Roche cobas c702 (Roche Diagnostics, Basel, Switzerland) which required 2 µL of plasma. Quantification of HSA had a turn-around-time of <3 h. This method has been standardized against the reference preparation of the IRMM (Institute for Reference Materials and Measurements) BCR470/CRM470 (RPPHS - Reference Preparation for Proteins in Human Serum).^18^ The assay’s measurement range was 2–60 g/L, with values > 60 g/L automatically diluted with 0.9% NaCl using a 1:3 dilution factor. The analytical variation of the test was 2.7% at 27.6 g/L and 1.9% at 45.0 g/L, with a lower limit of detection of 2 g/L. The assay used in UZL was BELAC (Belgian Accreditation body) accredited according to the ISO 15189 standard.^19^ Additional details on other laboratory tests are summarized in Supplementary Table S1.

### Statistical analysis

Statistical analyses were performed using SAS software (version 9.4, SAS System for Windows). Concentrations of HSA over time were modeled using linear mixed effects modeling. Due to drop-out in the longitudinal dataset, maximum likelihood estimation was used to flexibly deal with missing data.^20^ This method provides valid parameter estimates under a missing at random mechanism, implying that correct inference is possible even if missingness is related to model covariates or previous observations.^21^ Although data become sparser towards PNA 28 days, the estimated longitudinal profiles represent all patients in the cohort at all follow-up times without data imputation. This decision was based on a low missingness rate of baseline covariates (<7%) and the dataset’s large size. Polynomials were used to flexibly model non-linear evolutions over time.

#### Clinical characteristics

Baseline clinical characteristics were described using mean, standard deviation of the mean (std), median, interquartile range (IQR) and range (minimum-maximum) for continuous variables, and incidence (count and percentage) for dichotomous variables. Longitudinal data were visually presented as estimated means with 95% confidence intervals over time.

#### Univariable analyses exploring HSA covariates

A *p*-value of <0.05 was considered statistically significant. A segmented regression approach was applied for PNA, dividing the follow-up time into early (PNA 1–7 days) and late (PNA > 7 days) neonatal life. The effect of PNA on HSA was estimated separately in both periods. Birth weight and GA were analyzed as both continuous and categorical variables. Classification of BW and GA was based on a guidance document of the Food and Drug Administration.^22^ Birth weight categories were extremely low BW ( < 1000 g), very low BW (1000–1499 g), low BW (1500–2499 g), and BW ≥ 2500 g. Gestational age categories were extremely preterm (<28w), very preterm (28–<32w), moderate to late preterm (32–<37w), and full term (37–42w). To compare estimated mean HSA concentrations and mean difference between BW and GA categories, linear mixed models were used with post-hoc comparisons of least square means.

#### Multivariable HSA prediction model

To develop a multivariable HSA prediction model, a backward model selection procedure was followed with a 1% significance level for term elimination. First, higher order polynomials for continuous predictors were eliminated until none of the terms had *p*-values > 0.01. Second, main effects were eliminated in the same way. We did not include interactions with time in the model and removed parameter estimates ≤0.001 to maintain model simplicity and applicability. To quantify model performance, an internal validation was performed with a random sample of 700 patients from the full dataset. The model building procedure was performed exactly as in the actual model construction. The resulting model was used to make predictions for the remaining patients in the dataset. Model performance was quantified by R^2^, a metric for model performance. To describe the correlation between observed and predicted HSA values, an observed versus predicted and a Bland-Altman plot were created. Given the clustering in the longitudinal data, we calculated performance indices in two ways: (1) for all observations of the validation set, including repeated measures on subjects (R^2^ overall), and (2) per day and averaged over days (R^2^ day).

#### External validation of existing HSA prediction functions

A literature search was performed to screen for previously described neonatal HSA prediction functions. Model performance of available functions was explored by R^2^ and the correlation between observed HSA concentrations of our dataset and predicted HSA concentrations of the respective functions.

#### Illustration: impact of HSA on unbound vancomycin concentrations

To illustrate the impact of HSA on unbound drug concentrations, we predicted two sets of unbound vancomycin concentrations, a glycopeptide frequently used in neonates to treat gram-positive infections. First, concentrations were predicted for six virtual clinical cases (with a fixed total vancomycin concentration of 20 mg/L). Selected values for model covariates were based on mean values described in literature.^3,23–25^ We predicted HSA concentrations for each case using our HSA prediction function. Next, unbound vancomycin concentrations were calculated using the equation described by Smits et al.: unbound vancomycin concentration (mg/L) = 0.884 × total vancomycin concentration (mg/L) − 0.323 x HSA concentration (g/L) + 10.609.^13^ Second, the same exercise was performed for 6 real-world clinical cases (with observed HSA, total and unbound vancomycin concentrations available) of a previously published cohort on vancomycin protein binding in neonates.^13^ Observed versus predicted concentrations were compared and described.

### Ethics

The study was approved by the Ethics Committee Research UZ/KU Leuven (internal study number S61706) and conducted in compliance with the principles of the Declaration of Helsinki (current version, 2013), the principles of Good Clinical Practice (GCP), and all applicable regulatory requirements. Since the data in this study were collected retrospectively, informed consent was waived.

## Results

### Clinical characteristics

During the study period, 1053 neonates were admitted to the UZL NICU of whom 848 neonates were eligible for inclusion. The study population consisted of 394 female (46.5%) and 454 male (53.5%) neonates, with median (IQR) GA of 35 (32–38) weeks and BW of 2400 (1640–3130) grams. We collected 5548 HSA observations with a median of 5 (3–9) observations per patient. Median HSA concentration was 32.3 (28.7–35.6) g/L. Clinical characteristics of the included patients are provided in Table 1.

**Table 1:** Clinical characteristics of the included patients (*N* = 848).

Variable	Statistic	Result
*Number of HSA observations per patient N* *=* 848	Mean	6.54
	Std	5.37
	Median	5
	IQR	3.0–9.0
	Range	1.0–29.0
*Sex*		
Female	n/N (%)	394/848 (46.46%)
Male	n/N (%)	454/848 (53.54%)
*Gestational age (weeks) N = 843*	Mean	34.5
	Std	4.15
	Median	35.0
	IQR	32.0-38.0
	Range	23.0-42.0
*24-* < *28*	*n* (%)	57 (6.76%)
*28-* < *32*	*n* (%)	145 (17.20%)
*32-* < *37*	*n* (%)	316 (37.49%)
*37-* < *42*	*n* (%)	325 (38.55%)
*Birth weight (grams) N = 848*	Mean	2392.2
	Std	942.82
	Median	2400.0
	IQR	1640.0–3130.0
	Range	470.0–6000.0
*<1000*	*n* (%)	71 (8.37%)
*1000–1499*	*n* (%)	101 (11.91%)
*1500–2499*	*n* (%)	280 (33.02%)
*≥2500*	*n* (%)	396 (46.70%)
*Diagnosis group*		
Prematurity	n/N (%)	301/848 (35.50%)
Respiratory diagnosis	n/N (%)	184/848 (21.70%)
Cardiac diagnosis	n/N (%)	133/848 (15.68%)
Neurologic diagnosis	n/N (%)	94/848 (11.08%)
Gastro-intestinal diagnosis	n/N (%)	45/848 (5.31%)
Infection (confirmed/suspected)	n/N (%)	24/848 (2.83%)
Intra-uterine growth restriction	n/N (%)	23/848 (2.71%)
Hematological diagnosis	n/N (%)	23/848 (2.71%)
Metabolic/endocrine diagnosis	n/N (%)	11/848 (1.30%)
Nephro-urologic diagnosis	n/N (%)	5/848 (0.59%)
Other diagnosis	n/N (%)	5/848 (0.59%)

*n* number of patients, *N* total number of patients, *Std* standard deviation, *IQR* interquartile range.

### Longitudinal analyses

**Fig. 1:**
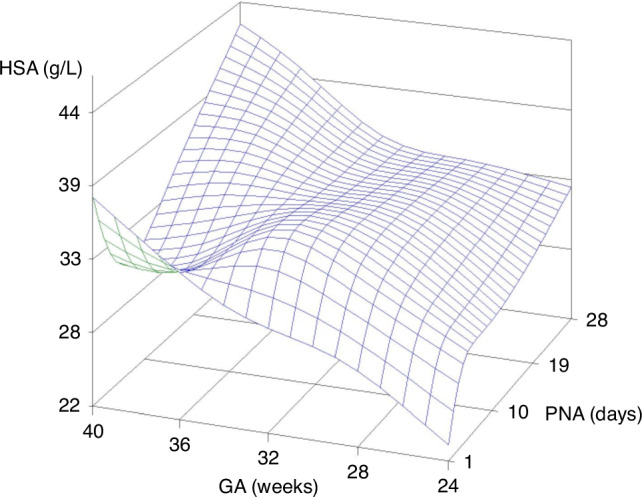
Estimated mean human serum albumin concentrations (g/L). Visual representation of the trends in HSA concentrations observed in this study by gestational age (weeks) and postnatal age (days): 3D plot. There is an increase in HSA concentrations with both GA and PNA, except for the most mature neonates where HSA concentrations decrease during the first postnatal week of life. HSA = human serum albumin (g/L); GA = gestational age (weeks); PNA = postnatal age (days).

Estimated centiles of HSA concentrations over PNA for the predefined GA categories are presented in Table 2, indicating an increase in HSA concentrations with both PNA and GA. Except for term neonates (GA 37- < 42 weeks), where HSA concentrations decreased during the first postnatal week and increased afterwards. In Fig. 1, estimated mean HSA values were 3D-plotted against GA and PNA for visual representation of the HSA concentration trends in the dataset.

**Table 2:** Estimated centiles (P5-25-50-75-95) of human serum albumin (g/L) over postnatal age (days) by gestational age (weeks).

GA (w)	Centile	Day 1	Day 2	Day 3	Day 4	Day 5	Day 6	Day 7	Day 14	Day 21
24- < 28 *n = 77*	P5	19.05	21.98	24.55	26.16	27.28	28.01	28.51	28.40	27.86
	P25	23.03	25.89	28.36	30.01	30.96	31.50	31.78	31.60	31.51
	**P50**	**25.18**	**28.00**	**30.32**	**31.90**	**32.80**	**33.27**	**33.53**	**33.15**	**32.89**
	P75	27.00	29.76	32.03	33.55	34.45	34.88	35.10	34.41	34.03
	P95	29.76	32.46	34.72	36.17	36.95	37.30	37.49	36.63	36.57
28- < 32 *n* = 174	P5	22.44	24.19	25.74	26.63	27.29	27.77	28.17	29.09	29.32
	P25	26.42	28.10	29.55	30.47	30.97	31.26	31.43	32.29	32.97
	**P50**	**28.57**	**30.21**	**31.51**	**32.36**	**32.81**	**33.03**	**33.19**	**33.84**	**34.36**
	P75	30.39	31.97	33.22	34.02	34.46	34.64	34.76	35.11	35.50
	P95	33.16	34.67	35.91	36.63	36.96	37.06	37.14	37.33	38.04
32- < 37 *n = 331*	P5	25.83	26.39	26.93	27.09	27.30	27.52	27.83	29.78	30.79
	P25	29.82	30.30	30.73	30.94	30.98	31.02	31.09	32.98	34.44
	**P50**	**31.96**	**32.41**	**32.70**	**32.83**	**32.82**	**32.79**	**32.85**	**34.53**	**35.82**
	P75	33.78	34.18	34.41	34.48	34.46	34.40	34.42	35.80	36.97
	P95	36.55	36.88	37.09	37.10	36.97	36.81	36.80	38.02	39.50
37- < 42 *n = 258*	P5	29.22	28.60	28.12	27.56	27.31	27.28	27.49	30.48	32.26
	P25	33.21	32.51	31.92	31.40	30.99	30.78	30.75	33.67	35.90
	**P50**	**35.35**	**34.62**	**33.89**	**33.29**	**32.82**	**32.55**	**32.51**	**35.22**	**37.29**
	P75	37.17	36.38	35.60	34.94	34.47	34.16	34.08	36.49	38.43
	P95	39.94	39.08	38.28	37.56	36.97	36.57	36.46	38.71	40.97

*GA* gestational age (weeks).

Bold values represent the median human serum albumin concentration for each GA category (g/L).

### Univariable analyses exploring HSA covariates

**Fig. 2:**
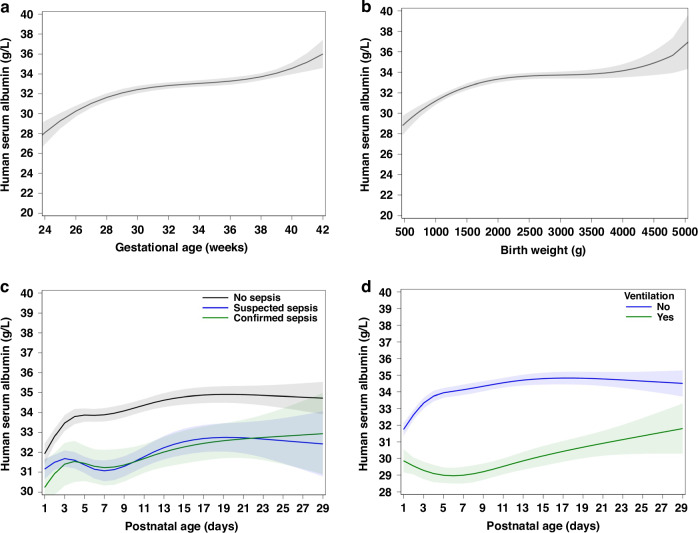
Univariable associations between covariates and human serum albumin. Association of human serum albumin with **a** gestational age, **b** birth weight, **c** sepsis, **d** mechanical ventilation. Full line = estimated means; shadow zone around full line = 95% confidence interval.

Univariable analyses revealed significant associations between HSA concentrations and PNA, GA, BW, CW, sepsis, total and direct bilirubin, total plasma proteins, respiratory support, mechanical ventilation, ibuprofen use, and CRP (*p*-values < 0.05, Supplementary Table S2). First order polynomials (linear trends) were selected for CW, total plasma proteins and total bilirubin, second-order polynomials for CRP, creatinine and direct bilirubin, and third-order polynomials for GA and BW. Supplementary Table S3 provides further exploration of GA and BW as categorical covariates. The difference in estimated HSA with the lowest GA (24-28w) and BW (<1000 g) category was explored, as well as the difference with the respective former category of GA or BW. There was a statistically significant difference between each two separate categories, except for BW ≥ 2500 g versus BW 1500–2499 g. Figure 2 visually presents the univariable associations of GA, BW, sepsis, and mechanical ventilation with HSA concentrations. Both presence of sepsis (*p* = 0.0011) and mechanical ventilation (*p* < 0.0001) were significantly associated with lower HSA concentrations.

### Multivariable HSA prediction model

**Fig. 3:**
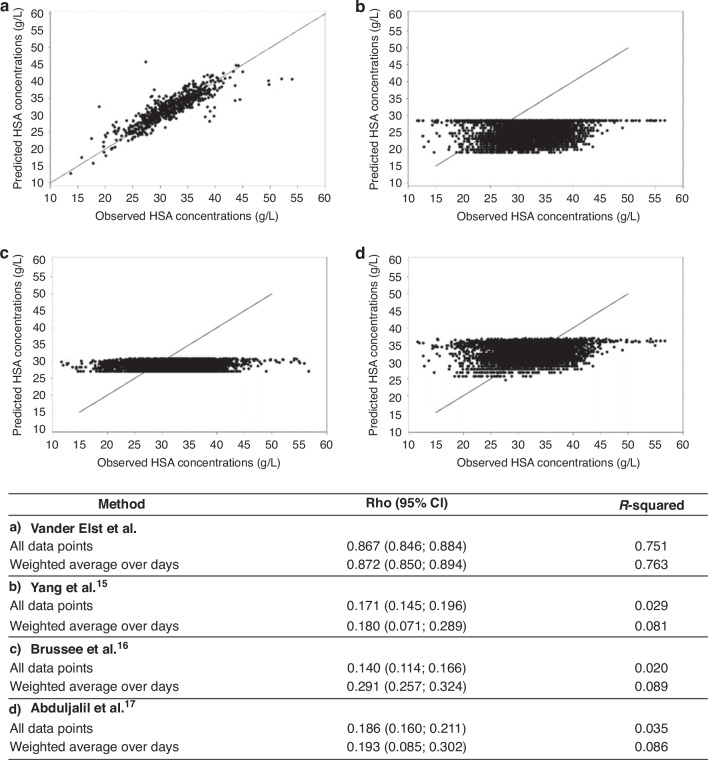
Observed versus predicted HSA concentrations. Model performance and scatterplot of observed HSA concentrations (g/L) of our dataset versus predicted HSA concentrations (g/L) of **a** our newly developed HSA prediction function, and of three published HSA prediction functions from **b** Yang et al.^15^ **c** Brussee et al.^16^ and **d** Abduljalil et al.^17^ HSA - human serum albumin (g/L); full line = line of unity; Rho = Pearson correlation; Weighted by the number of observation at each day.

Initially, following covariates were included in the model: PNA, GA, BW, CW, sex, admission diagnosis, sepsis, total and direct bilirubin, total plasma proteins, respiratory support, mechanical ventilation, ibuprofen use, serum creatinine, and CRP. Model development was based on 3736 observations from 792 patients, excluding 56 patients with missing data. Using a backward model selection, a HSA prediction model was developed containing 10 covariates with high model performance (R^2^ overall of 75.1% and R^2^ day of 76.3%, Fig. 3). The final model’s covariates are presented in Supplementary Table S4. Based on this model, the prediction function was:

HSA (g/L) = −0.177 - 0.126×PNA + 0.294×PNA2 - 0.197*GA + 0.401×BW + 0.896*total plasma proteins - 0.003×total plasma proteins^2^ + 0.126×total bilirubin + 0.941×direct bilirubin - 0.284×direct bilirubin^2^ + 0.017×direct bilirubin^3^ - 0.036×CRP + 5.447×serum creatinine - 2.748×serum creatinine^2^ + 0.380×serum creatinine^3^ - 0.562×ibuprofen use - 0.630×sepsis (with PNA2 = 0 if PNA < = 7; PNA2 = PNA-7 if PNA > 7).

In Fig. 3, observed HSA concentrations were plotted versus predicted concentrations. A Bland-Altman plot was created, demonstrating a mean difference of observed versus predicted HSA concentrations (which is a measure for bias of the model) of 0.24 g/L (Supplementary Table S5).

### External validation of three existing prediction functions

Three other neonatal HSA prediction functions were retrieved from literature:

1. HSA (g/dL) = (0.0805*PMA-0.102) for PMA ≥ 25 and <36.55 weeks
  HSA (g/dL) = (0.0025*PMA + 2.7506) for PMA ≥ 36.55 and <65 weeks^15^
2. HSA (g/dL) = 1.1287*ln(age[yr])+33.746 for GA 26–34 weeks^16^
3. HSA (g/dL) = (41.3*PMA^2.70^)/(0.383^2.70^ + PMA^2.70^) for GA 28–36 weeks^17^

Model performance was lower compared to model performance of our newly developed HSA prediction function, with R^2^ ranging from 2% to 8.9% (Fig. 3). In Fig. 3, observed HSA concentrations of our dataset were plotted versus predicted concentrations using these three functions.

### Illustration: impact of HSA on unbound vancomycin concentrations

Predicted HSA and unbound vancomycin concentrations for the six virtual cases are presented in Table 3. Predicted unbound vancomycin concentrations increased with decreasing predicted HSA concentrations. Substantial variability in unbound vancomycin concentrations was observed between the six virtual cases. Observed and predicted HSA and unbound vancomycin concentrations of the 6 real-world clinical cases demonstrated adequate observed versus predicted agreement (Supplementary Table S6).

**Table 3:** Variability in predicted human serum albumin (HSA) concentrations and its impact on predicted unbound vancomycin concentrations, as illustrated by 6 virtual clinical cases.

	Case 1	Case 2	Case 3	Case 4	Case 5	Case 6
	Extremely preterm	Moderate preterm	Term neonate	Extremely preterm with PDA	Moderate preterm with sepsis	Term neonate with hyperbilirubinemia
Gestational age (w)	25	32	40	25	32	40
Birth weight (g)	600	1750	3500	600	1750	3500
Postnatal age (d)	5	5	5	5	5	5
Total plasma proteins (g/L)	38	43	52	38	43	52
Total bilirubinemia (mg/dL)	5	9	16	5	9	23
Direct bilirubinemia (mg/dL)	0.15	0.25	0.55	0.15	0.25	0.65
C-reactive protein (mg/L)	2	4	1	5	95	2
Serum creatinine (mg/dL)	0.76	0.52	0.45	1.05	0.52	0.45
Ibuprofen use	0	0	0	1	0	0
Sepsis	0	0	0	0	1	0
**Predicted HSA (g/L)**	**27.15**	**29.01**	**34.23**	**27.05**	**25.2**	**35.13**
**Predicted unbound VAN (mg/L)**	**19.52**	**18.92**	**17.23**	**19.55**	**20.15**	**16.94**

*HSA* human serum albumin concentration (g/L), *VAN* vancomycin concentration (mg/L); Ibuprofen use: 1 = yes, 0 = no; Sepsis: 1 = yes, 0 = no

A fixed postnatal age of 5 days was selected, combined with increasing gestational age (case 1, 2, 3). For cases 4, 5, and 6 gestational age and birth weight were repeated and a disease setting of respectively patent ductus arteriosus (PDA, case 4), sepsis (case 5), and hyperbilirubinemia (case 6) was added (orange boxes). Selected total plasma proteins and serum creatinine values were based on mean values as described in literature.^3,27–29^ Predicted unbound vancomycin concentrations were calculated as described by Smits et al. ^13^ Total vancomycin concentration was fixed at 20 mg/L.

## Discussion

A high-performance HSA prediction model was developed for preterm and term neonates, incorporating both maturational and non-maturational covariates, to incorporate in future physiologically based PK (PBPK) and PK-PD research initiatives. In addition, HSA centiles based on PNA and GA groups were developed for application in clinical practice.

Our dataset demonstrated a progressive increase of HSA concentrations with both PNA and GA, aligning with available literature. In 1990, Reading et al. revealed increasing HSA concentrations with PNA and GA until PNA of 8 weeks in preterm neonates (<35 weeks GA).^6^ Kanakoudi et al. analyzed HSA concentrations in healthy infants (GA 26-41 weeks) at specific time points describing a progressive increase in HSA concentrations until 6 months of age.^5^ In a large retrospective cohort of 164 401 preterm and term neonates (GA 23–42 weeks) with a PNA of <14 days, Watchko et al. demonstrated that both PNA and GA influenced HSA concentrations significantly.^9^ The lowest HSA concentrations were observed during the first postnatal week of life. However, after 35 weeks GA, HSA concentrations seemed to reach a plateau which is different from the described trends in our study. Overall, the observed HSA concentrations in our dataset were higher compared to the data of Watchko et al. The fact that HSA concentrations are regularly measured as part of standard clinical care in the UZL NICU, might in part explain this difference. As a result, the dataset used for this study also contains HSA observations from less critically ill neonates, potentially resulting in higher HSA concentrations. Additional hypotheses for this difference might involve different approaches to fluid management and variations in albumin assay techniques. We could not find this information in the paper by Watchko et al. In this study, a colorimetric assay with bromocresol green (BCG) was used to determine HSA concentrations. The assay with bromocresol purple typically gives lower values compared to BCG.^26,27^ At least, this illustrates the caution needed when extrapolating results and emphasizes the need for external validation. Innovative in this retrospective analysis is the development of HSA centiles, which can be applied in clinical practice when interpreting HSA concentrations. These centiles illustrate a HSA increase with PNA and GA, except for term neonates. The initial HSA decrease in term neonates is probably a result of their admission because of severe illnesses (e.g. infections) or a post-surgical setting, known to lower HSA concentrations.^1,10,11^ HSA is a negative acute phase protein with reduced gene transcription during sepsis or hepatic disease, resulting in lower concentrations.^1,4^ Furthermore, during critical illness, cytokines often increase capillary permeability, resulting in HSA leakage into the interstitial space.^1^ Consequently, these neonates do not represent the ‘healthy’ term neonate, and centiles may not simply be extrapolated to the general neonatal population. Moreover, neonates with severe hypoalbuminemia often receive albumin infusions. Although no standardized neonatal guidelines exist, our unit uses a lower threshold of 25 g/L.

Our analysis utilized a detailed dataset combining neonatal clinical and laboratory real-world data. We investigated multiple covariates of interest for their potential application in future PK(-PD) research initiatives. Maturational covariates with a significant impact on neonatal HSA were PNA, GA, BW and CW. Significant non-maturational covariates were total and direct bilirubin, total plasma proteins, respiratory support, ibuprofen use, creatinine, and CRP. Moreover, both presence of sepsis and mechanical ventilation as non-maturational covariates were associated with significantly lower HSA concentrations, reflecting the lower HSA concentrations in critically ill neonates. This is important, as these patients are often exposed to multiple drugs and these covariates could be of relevance to improve future neonatal PK models of HSA-bound drugs.

Pharmacokinetic models, e.g. PBPK and population PK(-PD) models, are increasingly used to improve pharmacotherapy in neonates. To optimize these models, understanding neonatal physiology is crucial. The predictive performance of neonatal PK models is limited due to the rapidly changing and time-dependent physiology of neonates. Factors like kidney function (for renal drug clearance), hepatic function (for metabolic drug clearance), but also protein-binding of drugs (for drug distribution), are important to understand neonatal PK. Consequently, there is a clear and urgent need for neonatal physiology data to improve these PK models. To incorporate HSA trends in PK analyses, we therefore developed an accurate HSA prediction function. We are aware of three other neonatal HSA prediction functions previously described.^15–17^ However, their predictive performance was considerably lower (R^2^ 2–8.9%) compared to performance of our newly developed HSA prediction function. Our function’s strength is the integration of both maturational and non-maturational covariates, providing more accurate HSA predictions. Although we are aware of the complexity of the model, it clearly demonstrates its scientific relevance and added value to future PBPK and PK-PD modeling initiatives.

Unbound drug concentrations are responsible for drug (side-)effects and are influenced by HSA concentrations. For example, adverse effects of diazepam have been attributed to lower HSA concentrations resulting in higher unbound drug fractions in neonates.^3^ Conform, higher unbound drug fractions of vancomycin (90%) and cefazolin (40%) have been reported in neonates compared to older populations.^13,28,29^ However, drug dosing is still mostly based on total concentrations for both drugs. To illustrate the impact of HSA on unbound drug concentrations, we predicted unbound vancomycin concentrations for six virtual and six real-world clinical cases. Substantial variability in unbound vancomycin concentrations was observed between the six virtual cases which may cause sub- or supratherapeutic vancomycin exposure. The adequate observed versus predicted agreement of the six real-world clinical cases results in comparable clinical decisions or implications, illustrating the applicability of our prediction model. These examples emphasize the relevance of using unbound drug concentrations instead of total concentrations to guide dosing.

This study has its limitations. First, it concerns a single-center, retrospective analysis with internal validation to assess the performance of the developed model. This approach raises uncertainties regarding the generalizability of the results. To address this limitation, future external model validation is of relevance to evaluate model performance beyond our NICU population. Second, admission diagnosis relied on the clinicians’ diagnosis in the discharge report, lacking a standardized classification system for hospital diagnoses (e.g. International Classification of Diseases and Related Health Problems, ICD-10). Therefore, further sub-analyses for specific neonatal disease subgroups or specific congenital anomalies were not performed. However, this might further improve PK model performances. Third, information on the administration of blood products, total parenteral nutrition or hypoalbuminemia treatment with albumin infusions were not incorporated in the dataset. Consequently, we cannot exclude possible confounding of these covariates. Fourth, it is possible that HSA concentrations were more frequently determined in critically ill neonates, potentially introducing bias in the results. However, 88.8% of included neonates in our dataset had at least two HSA observations. Fifth, using serum creatinine as a surrogate marker for renal function has its limitations in neonates. Serum creatinine concentrations are influenced by multiple factors such as age, weight and muscle mass, and they reflect maternal serum creatinine concentrations in the first few days of life.^30^ Since more accurate and validated surrogate markers for renal function are not yet available at the bedside for this population, we chose to include serum creatinine as a covariate during model development. Finally, extremely low BW and extremely preterm neonates constituted the smallest group in the dataset (8.4% and 6.8%, respectively). Therefore, our results for this specific subgroup should be interpreted with caution. The strengths of this study include the use of real-world longitudinal data from a large cohort of (pre-)term neonates, and a thorough statistical analysis with the development of an accurate HSA prediction model. We intend to validate this model with an external dataset to enable extrapolation of study results. A next step would be to integrate our HSA prediction model, together with drug HSA-binding data, into neonatal PBPK models to further improve drug dosing of HSA-bound drugs in neonates.

## Conclusion

Human serum albumin is an important plasma protein for binding and transporting drugs. Based on a large real-world longitudinal dataset of preterm and term neonates, HSA trends and centiles were retrieved for application in clinical practice, and an accurate HSA prediction model was developed. This model is the first model to integrate both maturational and non-maturational covariates, resulting in accurate predictions of HSA concentrations in neonates (R^2^ day of 76.3%). By better capturing time-dependent physiology in neonates, our data might improve the predictive performance of neonatal PBPK models. Additionally, these results can be integrated in future PK-PD analyses to improve pharmacotherapy of HSA-bound drugs in neonates.

## Supplementary information

SupplementaryMaterial

## Data Availability

Data are available upon reasonable request to the corresponding author.
