# Supplementary material for: Human serum albumin: prediction model and reference values for preterm and term neonates
Source: Pediatr Res. 2024 Oct 11;99(2):604–11. doi: 10.1038/s41390-024-03634-1 (PMC12956593; doi:10.1038/s41390-024-03634-1)
Supplement: Supplementary file 1 — SupplementaryMaterial [file 41390_2024_3634_MOESM1_ESM.pdf]

Supplementary Material

Supplementary Table S1: Details on sampling and bio-analytical information of the laboratory covariates included in the dataset.

**Laboratory Value**	**Total plasma proteins**	**Total bilirubin**	**Direct bilirubin**	**Serum creatinine**	**C-reactive protein**
**Sample details***	Lithium heparin plasma	Lithium heparin plasma	Lithium heparin plasma	Lithium heparin plasma	Lithium heparin plasma
**Storage temperature before analysis**	Room temperature	Room temperature	Room temperature	Room temperature	Room temperature
**Centrifugation**	Room temperature, 10 min. at 2000 g	Room temperature, 10 min. at 2000 g	Room temperature, 10 min. at 2000 g	Room temperature, 10 min. at 2000 g	Room temperature, 10 min. at 2000 g
**Laboratory turn-around time (h)**	3	3	3	3	3
**Assay and reference number**[$]	Reagent TP2, ref. no. 05171385	Reagent BILT3, ref. no. 05795419	Reagent BILD2, ref. no. 05168384	Reagent CREP2, ref. no. 05168589	Reagent CRP4, ref. no. 07876424
**Analyzer**[$]	Cobas c702	Cobas c702	Cobas c702	Cobas c702	Cobas c702
**Required sample volume (µL)**	2	2	6.7	2	2
**Standardization**	Reagent TP2, ref. no. 05171385	Standardized against Doumas method[1]	Standardized against Jendrassik Grof method[2]	Standardized against ID/MS	Standardized against IRMM ERM/DA474/IFCC[3]
**Range reported by the laboratory**	2 - 360 g/L, 1/3 dilution with 0.9% NaCl for values > 120 g/L	0.18 - 76 mg/dL, 1/2.37 dilution with 0.9% NaCl for values > 32.2 mg/dL	0.18 - 34 mg/dL, 1/2 dilution with 0.9% NaCl for values > 17 mg/dL	0.06-122.00 mg/dL, 1/4 dilution with 0.9% NaCl for values > 30.5 mg/dL	0.6-700 mg/L, 1/2 dilution with 0.9% NaCl for values > 350 mg/L
**Analytical variation (% at concentration)**	1.55% at 42.3 g/L<br>1.32% at 67.6 g/L	2.65% at 1.117 mg/dL<br>1.25% at 4.117 mg/dL	3.08% at 0.550 mg/dL<br>2.29% at 1.546 mg/dL	2.02% at 0.894 mg/dL<br>1.49% at 5.970 mg/dL	2.54% at 7.48 mg/L<br>2.44% at 44.90 mg/L
**Assay LoQ**	2 g/L	0.146 mg/dL	0.18 mg/dL	0.06 mg/dL	0.6 mg/L

*All samples taken in Minicollect Lithium heparin (Greiner Bio-One, Kremsmünster, Austria); [$]All from Roche Diagnostistics (Basel, Switzerland); LoQ = lower limit of quantification

[1]Doumas, B. T. et al. Candidate Reference Method for Determination of Total Bilirubin in Serum: Development and Validation. *Olin Chem* **31**, 1779-1789 (1985).

[2]Jendrassik, L. & Grof, P., Vereinfachte photometrische Methode zur Bestimmung des Bilirubins. *Biochem Z* **297**, 81-89 (1938).

[3]Auclair, G. et al. CERTIFICATION REPORT. The Certification of the Mass Concentration of C-Reactive Protein in Human Serum. Publications Office of the European Union (2011).

Supplementary Table S2: Univariable analyses exploring the impact of covariates on human serum albumin concentrations in neonates.

Covariate	Effect	Estimate (95% CI)	P-value
Postnatal age	Global test		<.0001
	Slope (1-7 days)	0.244 (0.170;0.318)	<.0001
	Slope (>7 days)	0.081 (0.042;0.119)	<.0001
Gestational age	Global test / non-linear trend		<.0001
	26-30 weeks	2.172 (1.593;2.751)	<.0001
	30-35 weeks	0.726 (0.238;1.214)	0.0036
	35-40 weeks	1.410 (0.679;2.141)	0.0002
Birth weight	Global test / non-linear trend		<.0001
	1000-1500 grams	1.381 (1.047;1.715)	<.0001
	2000-2500 grams	0.307 (0.124;0.491)	0.0010
	3000-3500 grams	0.100 (-0.143;0.344)	0.4206
Current weight	+1000 grams (linear effect)	0.691 (0.461;0.920)	<.0001
Sepsis	Global test		<.0001
	Confirmed sepsis vs suspected sepsis	-0.425 (-1.044;0.193)	0.1780
	Confirmed sepsis vs no sepsis	-2.471 (-3.046;-1.895)	<.0001
	Suspected sepsis vs no sepsis	-2.046 (-2.328;-1.763)	<.0001
Ibuprofen	Yes vs No	-0.972 (-1.568;-0.376)	0.0014
Respiratory support	Yes vs No	-2.713 (-3.021;-2.405)	<.0001
Ventilation	Yes vs No	-3.967 (-4.327;-3.608)	<.0001
C-reactive protein	Global test / non-linear trend		<.0001
	5 – 15 mg/L	-1.450 (-1.703;-1.197)	<.0001
	15 – 25 mg/L	-0.672 (-0.766;-0.578)	<.0001
Serum creatinine	Global test / non-linear trend		<.0001
	0.5 – 1 mg/dL	0.569 (0.197;0.941)	0.0027
	1 - 1.5 mg/dL	-0.391 (-0.799;0.017)	0.0601
Total plasma proteins	+1 g/L (linear effect)	0.597 (0.588;0.607)	<.0001
Total bilirubinemia	+1 mg/dL (linear effect)	0.303 (0.264;0.342)	<.0001
Direct bilirubinemia	Global test / non-linear trend		<.0001
	0.5 – 1 mg/dL	1.639 (1.321;1.957)	<.0001
	1 - 1.5 mg/dL	0.177 (0.023;0.330)	0.0242

CI = confidence interval; Slope = estimated change in albumin for a 1-day increase of PNA; Estimate >(<)0 means higher (lower) human serum albumin level for first category (categorical variables) or highest level (continuous variables)

Supplementary Table S3: Univariable analyses exploring the impact of GA and BW as categorical variables on human serum albumin concentrations in neonates.

	Estimated HSA (g/L)	Difference vs [24-28w]		Difference vs former category	
GA	Mean (95% CI)	Mean difference (95% CI)	P-value	Mean difference (95% CI)	P-value
24-<28w	30.2 (29.5;30.9)		.		.
28-<32w	32.1 (31.6;32.5)	1.8 (1.0;2.7)	<.0001	1.8 (1.0;2.7)	<.0001
32-<37w	33.1 (32.8;33.5)	2.9 (2.1;3.7)	<.0001	1.1 (0.5;1.6)	0.0004
37-<42w	34.0 (33.6;34.4)	3.8 (3.0;4.6)	<.0001	0.9 (0.4;1.5)	0.0011
	**Estimated HSA (g/L)**	**Difference vs [<1000g]**		**Difference vs former category**	
**BW**	**Mean (95% CI)**	**Mean difference (95% CI)**	**P-value**	**Mean difference (95% CI)**	**P-value**
<1000g	30.1 (29.5;30.7)				
1000-1499g	32.2 (31.7;32.7)	2.1 (1.3;2.9)	<.0001	2.1 (1.3;2.9)	<.0001
1500-2499g	33.4 (33.0;33.8)	3.3 (2.6;4.0)	<.0001	1.2 (0.6;1.9)	0.0003
≥2500g	33.7 (33.3;34.0)	3.5 (2.8;4.2)	<.0001	0.2 (-0.3;0.8)	0.3806

GA = gestational age (weeks); BW = birth weight (g); HSA = human serum albumin (g/L); CI = confidence interval;
Global effect of GA: p=<.000; Global effect of BW: p=<.0001

Supplementary Table S4: Covariates included in the human serum albumin prediction model based on a multivariable analysis of our neonatal dataset.

Effect	Estimate	Standard error	RSE (%)
Intercept	-.176995	1.209499	-683.352
PNA	-.126300	0.026671	-21.117
PNA2	0.293556	0.032027	10.910
GA	-.197138	0.031994	-16.229
BW	0.400995	0.140805	35.114
Prot	0.895963	0.036974	4.127
$Prot^2$	-.003391	0.000381	-11.250
TotalBili	0.125969	0.012759	10.129
DirectBili	0.941015	0.196569	20.889
$DirectBili^2$	-.283729	0.068327	-24.082
$DirectBili^3$	0.016933	0.004681	27.642
CRP	-.036381	0.002371	-6.517
Creat	5.446667	0.755870	13.878
$Creat^2$	-2.74752	0.514615	-18.730
$Creat^3$	0.380053	0.087427	23.004
IBU	-.562447	0.171164	-30.432
Sepsis	-.630236	0.078748	-12.495

Model based on 3736 observations and 792 patients; PNA2=0 if PNA<=7; PNA2=PNA-7 if PNA>7; ^{2} refers to the second power, ^{3} refers to the third power; PNA = postnatal age (days); GA = gestational age (weeks); BW = birth weight (g); Prot = total plasma proteins (mg/dL); TotalBili = total bilirubin (mg/dL); DirectBili = direct bilirubin (mg/dL); CRP = C-reactive protein (mg/L); Creat = serum creatinine; IBU = ibuprofen use

Supplementary Table S5: Bland-Altman plot of the average of predicted and observed HSA concentrations (g/L) versus the difference between predicted and observed HSA concentrations (g/L).

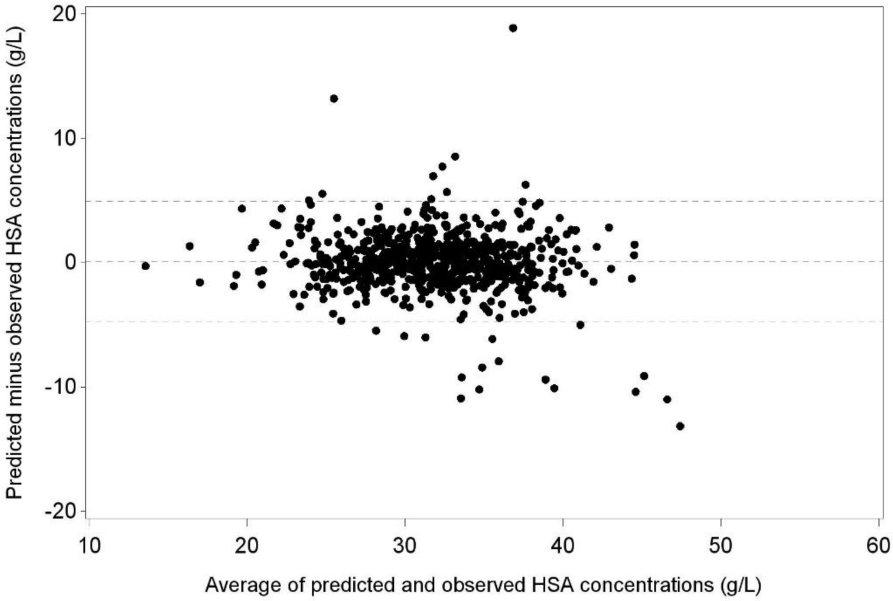

Difference (g/L)				95% LOA (g/L)		Direction		Magnitude	
Mean	SD	Median	P-value	Lower	Upper	Rho	P-value	Rho	P-value
0.24	2.54	0.25	0.001	-4.72	5.21	-0.15	<.001	0.04	0.299

HSA = human serum albumin (g/L); SD = standard deviation; P = p-value signed rank test; LOA = limits of agreement

Supplementary Table S6: Predicted human serum albumin (HSA) concentrations and its impact on predicted unbound vancomycin concentrations compared to observed HSA concentrations and observed unbound vancomycin concentrations, as illustrated by 6 real-world clinical cases. Case selection from a previously published cohort was based on availability of model covariates and variability in gestational age.[13] Predicted unbound vancomycin concentrations were calculated as described by Smits et al. and based on predicted HSA concentrations.[13]

	Case 1	Case 2	Case 3	Case 4	Case 5	Case 6
	Extremely preterm	Very preterm	Very preterm	Late preterm	Term	Term
**Gestational age (weeks)**	27	30	31	36	37	40
**Birth weight (g)**	893	924	1860	3000	2500	4425
**Postnatal age (days)**	11	10	6	10	5	8
**Total plasma proteins (g/L)**	38	52	56	43	36	49
**Total bilirubinemia (mg/dL)**	6.57	4.41	7.04	8.28	10.87	0.68
**Direct bilirubinemia (mg/dL)**	0.52	0.41	1.08	0.79	0.41	0.18
**C-reactive protein (mg/L)**	14.9	28.3	1.2	2.2	17.4	10.6
**Serum creatinine (mg/dL)**	0.99	0.46	0.64	0.28	0.36	0.34
**Ibuprofen use**	0	0	0	0	0	0
**Sepsis**	1	1	1	1	1	1
**Total VAN (mg/L)**	5.41	4.06	9.18	8.64	49.48	17.62
**Predicted HSA (g/L)**	27.31	32.46	36.79	27.72	22.56	29.12
**Observed HSA (g/L)**	23.6	30.6	38.1	29.9	24.2	27.6
**Predicted unbound VAN (mg/L)**	6.57	3.71	6.84	9.29	47.06	16.78
**Observed unbound VAN (mg/L)**	5.15	4.05	7.85	8.43	45.30	13.53

HSA = human serum albumin concentration (g/L); VAN = vancomycin concentration (mg/L); Ibuprofen use: 1 = yes, 0 = no; Sepsis: 1 = yes, 0 = no
